# Bacterial translocation and *in vivo* assessment of intestinal barrier permeability in rainbow trout (*Oncorhynchus mykiss*) with and without soyabean meal-induced inflammation

**DOI:** 10.1017/jns.2016.7

**Published:** 2016-06-06

**Authors:** Peyman Mosberian-Tanha, Margareth Øverland, Thor Landsverk, Felipe E. Reveco, Johan W. Schrama, Andries J. Roem, Jane W. Agger, Liv T. Mydland

**Affiliations:** 1 Department of Animal and Aquacultural Sciences, Norwegian University of Life Sciences, PO Box 5003, NO-1432 Ås, Norway; 2 Department of Basic Sciences and Aquatic Medicine, Norwegian University of Life Sciences, N-0033 Oslo, Norway; 3 Aquaculture and Fisheries Group, Wageningen Institute of Animal Sciences, PO Box 338, 6700 AH Wageningen, The Netherlands; 4 Department of Chemistry, Biotechnology and Food Science, Norwegian University of Life Sciences, PO Box 5003, NO-1432 Ås, Norway

**Keywords:** Rainbow trout, Soyabean meal, Enteritis, Intestinal permeability, Permeability markers, DGGE, denaturing gradient gel electrophoresis, DI, distal intestine, FCR, feed conversion ratio, FM, fish meal, L:R, lactulose:l-rhamnose, S:R, sucralose:l-rhamnose, SBM, soyabean meal, SBMIE, soyabean meal-induced enteritis

## Abstract

The primary aim of this experiment was to evaluate the intestinal barrier permeability *in vivo* in rainbow trout (*Oncorhynchus mykiss*) fed increasing levels of soyabean meal (SBM). The relationship between SBM-induced enteritis (SBMIE) and the permeability markers was also investigated. Our results showed that the mean score of morphological parameters was significantly higher as a result of 37·5 % SBM inclusion in the diet, while the scores of fish fed 25 % SBM or lower were not different from those of the fish meal-fed controls (*P* < 0·05). SBMIE was found in the distal intestine (DI) in 18 % of the fish (eleven of sixty): ten in the 37·5 % SBM-fed group and one in the 25 % SBM-fed group. Sugar markers in plasma showed large variation among individuals probably due to variation in feed intake. We found, however, a significant linear increase in the level of plasma d-lactate with increasing SBM inclusion level (*P* < 0·0001). Plasma concentration of endotoxin was not significantly different in groups with or without SBMIE. Some individual fish showed high values of endotoxin in blood, but the same individuals did not show any bacterial translocation. Plasma bacterial DNA was detected in 28 % of the fish with SBMIE, and 8 % of non-SBMIE fish (*P* = 0·07). Plasma concentration of d-lactate was significantly higher in fish with SBMIE (*P* < 0·0001). To conclude, SBMIE in the DI of rainbow trout was associated with an increase in bacterial translocation and plasma d-lactate concentration, suggesting that these permeability markers can be used to evaluate intestinal permeability *in vivo*.

Inclusion of sustainable ingredients as substitutes of traditional fish meal (FM) in fish diet is crucial to ensure further growth in the aquaculture sector^(^^1^^)^. Plant protein ingredients can serve as potential alternatives to replace FM^(^^2^^)^. As a result, substitution of FM by these ingredients has been reported and discussed in several publications^(^^3^^–^^5^^)^. Plant proteins contain anti-nutritional factors which can cause nutritional and health issues in salmonid fish such as inflammatory response in the distal intestine (DI) and lowered macronutrient digestibility^(^^6^^–^^8^^)^. The most widely used plant protein in animal production is soyabean meal (SBM)^(^^2^^)^ which has also been studied in carnivorous fish. SBM, however, has shown to cause inflammation in the DI of salmonid fish, often referred to as SBM-induced enteritis (SBMIE)^(^^7^^)^, the cause of which is not yet fully known. SBMIE has been used as a model to study plant ingredient-induced enteropathy in salmonids^(^^8^^,^^9^^)^.

The gastrointestinal tract acts as a barrier between the external and internal environments. The integrity of the gut barrier is crucial to maintain homeostasis, that is, to prevent pathogens or toxins from entering the bloodstream while maintaining nutrient absorption function^(^^10^^,^^11^^)^. The barrier function of the gut is supported by epithelial cells, mucus and tight junction proteins^(^^12^^–^^14^^)^. Increased gut permeability due to the loss of barrier function potentiates systemic absorption of pathogens and toxic molecules which has been shown to be associated with intestinal inflammation in mammals and fish^(^^15^^,^^16^^)^.

Epithelial permeability function has been assessed in mammals by *in vitro* methods such as transepithelial electrical resistance and *in vivo* tests such as transepithelial passage of sugar markers, endotoxins and d-lactate^(^^13^^,^^14^^)^. The *in vivo* methods are based on the assumption that microbes, large molecules and bacterial products cannot pass through the epithelial barrier and be absorbed in blood when the intestinal integrity is maintained^(^^14^^)^. Urinary or plasma levels of orally administered sugars such as sucrose, mannitol, rhamnose, lactulose and sucralose have been used as markers in mammalian intestinal permeability evaluations^(^^17^^,^^18^^)^. In these studies the ratio of disaccharides to monosaccharides in samples are calculated and used to assess barrier function^(^^18^^)^. Degradability of sugar molecules in different regions of the gastrointestinal tract has led to region-specific permeability experiments^(^^13^^)^. For example, the lactulose:l-rhamnose ratio (L:R) has been used for small-intestinal permeability evaluation because lactulose is fermentable in the colon^(^^19^^)^. In humans, inflammatory bowel disease and coeliac disease are two examples of intestinal inflammation which have been shown to be associated with increased urinary levels of sugar markers^(^^20^^,^^21^^)^.

Blood or plasma measurement of bacteria and their products is another *in vivo* method used for the evaluation of bacterial translocation and intestinal permeability. Measurement of plasma endotoxin, d-lactate and detection of bacterial DNA using PCR-based methods have been used to assess the function of the intestinal barrier. Lipopolysaccharides are endotoxins which partially form the outer membrane of Gram-negative bacteria and are known to be toxic to humans and animals^(^^22^^,^^23^^)^. For quantitative measurement of endotoxin levels in plasma samples, the limulus amebocyte lysate assays (LAL), such as chromogenic LAL, have been widely used as a sensitive method^(^^24^^)^. Intestinal inflammation has shown to be associated with increased endotoxin levels in the circulation which could be a result of epithelial barrier hyperpermeability^(^^25^^,^^26^^)^. Many bacteria in the gastrointestinal tract produce d-lactate which has been used as a permeability marker in humans and animals due to the fact that it cannot be metabolised by mammals^(^^27^^–^^29^^)^. In healthy individuals, a low concentration of d-lactate is found in plasma, but the level is known to increase as a result of increased intestinal permeability^(^^14^^,^^30^^)^. PCR is a sensitive method which has been used for the detection of bacterial DNA in blood to assess intestinal barrier function in a number of experiments^(^^31^^–^^33^^)^. PCR has proven to be more sensitive than conventional culture methods in detecting bacteria and it is also able to detect dead bacteria in samples^(^^31^^,^^34^^)^.

The effect of plant ingredients on intestinal barrier permeability and function is not yet fully understood in fish. Experiments on intestinal barrier permeability in fish has been mainly based on *in vitro* and molecular studies such as studies of gene expression^(^^35^^–^^38^^)^. SBM or SBM-extracted alcohol-soluble substances are examples of dietary factors which have been shown to increase intestinal permeability in fish *in vitro*^(^^15^^,^^39^^)^ and alter morphology of brush borders leading to tight junction exposure to the luminal components^(^^40^^)^. To our knowledge, there is no information available on *in vivo* assessment of intestinal permeability in salmonids fed SBM. Thus, this experiment was designed to evaluate intestinal barrier permeability *in vivo* in rainbow trout fed with increasing levels of SBM by using plasma endotoxin, d-lactate, sugar molecules and detection of bacterial DNA as markers. Furthermore, the change in intestinal permeability was investigated in relation to SBMIE.

## Materials and methods

### Fish husbandry and experimental diets

The experiment was performed at the fish laboratory located at the Norwegian University of Life Sciences (Ås, Norway), which is an approved research facility by the Norwegian Animal Research Authority and operates in accordance with the Norwegian Regulations of 17 June 2008 no. 822: Regulations relating to Operation of Aquaculture Establishments (Aquaculture Operation Regulations). A total of 300 rainbow trout (*Oncorhynchus mykiss*) with mean initial body weight of 236 g were randomly allocated into twelve tanks. Each tank (300 litres) was supplied with recirculated fresh water at a flow rate of 7·5 litres/min under continuous light. Water temperature ranged from 8 to 11°C. Daily measurement of dissolved O_2_ levels was performed and kept above 8·0 mg/l in the outlet water. Four iso-energetic experimental diets were formulated and consisted of a FM-based control and three experimental diets containing SBM at levels of 12·5, 25 and 37·5 % (Table 1). The total experimental period lasted for 31 d, and a mixture of sugar markers, consisting of 10 g lactulose, 10 g sucralose and 5 g l-rhamnose (Sigma-Aldrich), was added per kg of each diet and fed during the last 3 d. Fish were fed 30 % in excess and uneaten feed was collected from the tank outlets after each feeding period. Feed preparation and daily feed intake calculations were performed as described in Øverland *et al.*^(^^41^^)^. The fish were fed until the time of sampling to avoid an empty intestine in fish used for analysis.

**Table 1.** Formulation and chemical composition (as-is) of fish meal (FM) and experimental diets

	FM	SBM 12·5 %	SBM 25 %	SBM 37·5 %
Ingredients (g/kg, as-fed)				
Fish meal*	620·0	500·0	370·0	229·0
Soyabean meal†	–	125·0	250·0	375·0
Fish oil‡	183·9	178·9	183·9	199·9
Gelatinised potato starch§	110·0	110·0	110·0	110·0
Gelatin‖	80·0	80·0	80·0	80·0
Premix¶	6·0	6·0	6·0	6·0
Y_2_O_3_**	0·1	0·1	0·1	0·1
Analysed content				
DM	950	935	927	944
Crude protein (g/kg DM)	512	489	462	432
Crude lipid (g/kg DM)	240	229	256	227
Starch (g/kg DM)	112	115	108	116
Ash (g/kg DM)	109	95	83	69
Gross energy (MJ/kg DM)	23·1	23·0	23·2	23·2

SBM, soyabean meal.

* NorseaMink.

† Denofa.

‡ Skretting Norway.

§ Lygel F 60, Lyckeby Culinar AB.

‖ Rousselot™ 250 PS, Rousselot SAS.

¶ Provided the following (per kg diet): Ca 1·2 g, Mn_2_SO_4_ 14·7 mg, ZnSO_4_ 117 mg, CuSO_4_ 4·90 mg, CoSO_4_ 980 µg, Ca(IO_3_)_2_ 3·6 mg, retinol 2450 IU, cholecalciferol 1470 IU, tocopherol 196 mg, menadione 9·80 mg, thiamine 14·7 mg, riboflavin 24·5 mg, pyridoxine 14·7 mg, cobalamine 19·6 µg, pantothenic acid 29·4 mg, folic acid 4·90 mg, niacin 73·5 mg, biotin 245 µg, vitamin C 1·75 (Rovimix^®^ Stay-C^®^ 35, DSM Nutritional Products), AS Norsk Mineralnæring.

** Di-yttrium trioxide (Y_2_O_3_) (Metal Rare Earth, Ltd).

### Fish sampling

On day 28, before the addition of sugar markers to the diets, nine fish per treatment (three fish/tank) were randomly selected and anaesthetised with Aquacalm (12 mg/l) for blood sampling. Blood samples were collected from the caudal vein in heparinised sterile syringes, transferred to Eppendorf tubes, and centrifuged (3000 ***g*** for 5 min) to obtain plasma. The plasma was then stored at −80°C for sugar marker analysis to determine any possible concentration of these molecules which was not due to the consumption of sugar marker-containing diets. After blood sampling fish were killed by a sharp blow to the head. At the end of the experiment and after feeding sugar-containing diets, five fish per tank were individually weighed and anaesthetised with Finquel (60 mg/l) prior to blood sampling, as described above. The anaesthetised fish were killed by a sharp blow to the head prior to dissection. Digesta samples from the DI were collected by carefully scraping with a sterile spatula into sterile containers, and stored on dry ice until transfer to −80°C. Tissue samples were also taken from the DI of each of the five fish and cut lengthways for morphological evaluation and fixed in neutral buffered formalin (4 % formaldehyde) for 48 h and then dehydrated in 70 % ethanol before embedding in paraffin following standard routines.

### Chemical analysis

Diets were analysed for DM by drying at 105°C to constant weight, ash by incineration at 550°C overnight, crude protein by the Kjeldahl method (N × 6·25), crude fat by HCl hydrolysis followed by diethyl ether extraction, starch by α-amylase and amyloglucosidase hydrolysis and gross energy by bomb calorimetry (Parr 1271 bomb calorimeter; Parr).

### Histology

Dehydrated DI tissues were embedded in paraffin and stained by haematoxylin and eosin (H&E) following a standard procedure. Blinded evaluation of four morphological parameters (Table 2) was performed on each tissue. A score was given to each parameter which ranged from 0 (no morphological change) to 2 (extreme changes). Scores of 0·5 and 1 were given to very slight changes (assessed as normal morphology) and mild changes, respectively. Based on this protocol at least a score of 1 should be given to the lamina propria, epithelial changes and atrophy to confirm occurrence of SBMIE. The total histological score was calculated by taking the average of the scores of the parameters to express the degree of SBMIE.

**Table 2.** Scoring system used to evaluate the degree of morphological changes in the distal intestine of soyabean meal-fed rainbow trout (*Oncorhynchus mykiss*)

Parameters	Score range	Description
Lamina propria	0–2	Leucocyte (e.g. lymphocyte, granulocytes and eosinophilic granular cells) infiltration and accumulation in the lamina propria
Epithelial changes	0–2	Reduced supranuclear vacuolisation Reduced height of epithelial cells Increased cytoplasmic basophilia
Atrophy	0–2	Shortening of the intestinal folds
Oedema	0–2	Accumulation of fluid in the lamina propria

### DNA extraction

Genomic DNA was extracted from plasma with the QIAamp DNA Blood Mini Kit (Qiagen) according to the manufacturer's protocol. Extracted DNA was then quantified and the purity was measured with a NanoDrop™ 8000 spectrophotometer (Thermo Scientific) before it was stored at −20°C for further analysis.

### Nested PCR protocol

Amplification of 16S rRNA genes from bacteria was performed using universal bacterial primers in a nested PCR. Primary amplification reactions were performed using primers 357F (5′-CCT AGG GGA GGC AGC AG-3′) and 1369R 5′-GCCCGGGAACGTATTCACCG-3′) in a 25 µl reaction mixture containing 500 nm of each of the primers, 90 ng of DNA template, 1 × reaction buffer, 1·75 mm-MgCl_2_, 300 µm of each deoxyribonucleotide triphosphate (dNTP), 1·25 U Platinum^®^ Taq DNA polymerase (Invitrogen) and 0·1 % bovine serum albumin. Negative controls (DNA-free water) were included in all sets of PCR reactions to provide a contamination check. Reaction mixtures were subjected to the following cycling conditions: 94°C for 4 min then 59°C for 60 s and 72°C for 90 s followed by seven cycles of touch down PCR (30 s at 94°C, 30 s with 1°C/cycle decrement from 59°C and 1 min at 72°C). This was further followed by thirty cycles of 94°C for 30 s, 53°C for 30 s and 72°C for 90 s with a final extension step of 10 min at 72°C. The secondary (nested) PCR was conducted using primers 357F (5′-CCT AGG GGA GGC AGC AG-3′) containing a 40-bp GC clamp at the 5′ end and 519R (5′-ATT ACC GCG GCK GCT GG-3′) in a 50 μl reaction mixture for amplification of the V3 region of the bacterial 16S rRNA genes. Nested PCR reaction mixtures contained: 200 µm of each dNTP, 1·75 mm-MgCl_2_, 500 nm of each primer, 1× PCR buffer, 1·25 U Platinum^®^ Taq DNA polymerase (Invitrogen) and PCR products from the first round. A re-amplified negative control from the first-round PCR and a new negative control using water were also included. PCR conditions were as follows: 4 min at 94°C, 1·5 min at 64°C and 1·5 min at 72°C followed by seven cycles of touchdown PCR (30 s at 94°C, 30 s with an 1°C/cycle decrement from 64°C and 60 s at 72°C) and followed by twenty-seven cycles of 45 s at 94°C, 45 s at 58°C, 60 s at 72°C, and a final extension step of 10 min at 72°C. To confirm successful amplification, 8 µl of PCR products from each amplification step were analysed by gel electrophoresis (1·5 % agarose, stained with RedSafe™ Nucleic Acid Staining Solution from iNtRON Biotechnology, Inc.), and images were taken using the Gel Doc XR System (Bio-Rad Laboratories).

### Denaturing gradient gel electrophoresis analysis

Denaturing gradient gel electrophoresis (DGGE) was performed to separate PCR products of 16S rRNA genes. Polyacrylamide gels (7·5 % (w/v) acrylamide) were made according to the manufacturer's instructions and were run on an Ingeny PhorU apparatus (Ingeny International BV) in a 0·5× TAE buffer (containing Tris base, acetic acid and EDTA). Denaturing gradients ranged from 42 to 58 % (where 100 % is defined as 7 m-urea and 40 % (v/v) formamide). Electrophoresis was performed at 75 V for 16 h at 60°C and gels were stained with SYBR Gold nucleic acid gel stain (Invitrogen) in 1× TAE for 10 min and photographed with Gel Doc XR System (Bio-Rad Laboratories).

### Denaturing gradient gel electrophoresis band sequencing

Selected DGGE bands were excised from the gel with sterile pipette tips. Each piece was then transferred into 50 µl of sterile water and eluted overnight at 4°C. Eluted DNA (3 µl) was subject to re-amplification applying the secondary (nested) PCR conditions as described previously, but with the following changes: eighteen cycles of PCR using 357F primer without the GC clamp and the volume of reaction was 25 µl. Amplification products were visualised as described previously prior to purification using the MultiScreen 96-well filtration plate (Millipore). Sequencing was carried out using the BigDye^®^ Terminator v3.1 Cycle Sequencing Kit, 357F and 519R primers on an ABI 3730 DNA analyser (Applied Biosystems). The BLAST (Basic Local Alignment Search Tool) program was used to search for the species with the closest known relationship with the 16S rRNA gene sequences.

### Biochemical assays

Endotoxin concentration in plasma samples was measured using ToxinSensor™ Chromogenic LAL Endotoxin Assay Kit (GenScript) under sterile conditions. The plasma samples were diluted at 1:1 (v/v) in endotoxin-free water and heated to 80°C for 10 min to remove non-specific endotoxin inhibitors. Endotoxin levels in samples were calculated from a standard curve of known endotoxin concentrations according to the manufacturer's instructions. Heparinised plasma was deproteinised by centrifugation through 10 K spin columns before the d-lactate concentration was measured using a commercial kit (d-lactate colorimetric assay; Sigma-Aldrich). The absorbance at 450 nm was read on a Wallac Victor 3 Multi-well Plate Reader (Perkin Elmer). d-Lactate concentration in distal intestinal content was determined using the same kit. In brief, an accurately weighed 200 mg sample of digesta was mixed well with two volumes (400 µl) of the d-lactate assay buffer. The digesta suspension was centrifuged through 10 K spin columns and the d-lactate concentration was assayed in 1:5 dilutions of the intestinal filtrate.

### Plasma analysis of sugar markers

Plasma was filtrated by centrifugation through a 10 K spin column and diluted 1:1 with water before analysis by high-performance anion exchange chromatography (HPAEC) using a Dionex ICS3000 connected to a CarboPac PA1 column (2 × 250 mm^2^) equipped with a guard of the same type (2 × 50 mm^2^) (Dionex). The HPAEC system was operated with a flow rate of 0·25 ml/min. Start eluent conditions was 10 % eluent A (0·1 m-NaOH) and 90 % eluent C (MQ-water) over 5 min, then 100 % A for 9 min, from 14 to 19 min an increase in eluent B (0·1 m-NaOH + 1 m-NaOAc) from 0 to 50 %, along with a decrease in eluent A from 100 to 50 %, and thereafter back to 10 % eluent A for 25 min. Eluted sugar markers (l-rhamnose, lactulose, sucralose) were monitored by the pulsed amperometric detector fitted with disposable gold working electrodes to increase the sensitivity, chromatograms were recorded using Chromeleon software (Dionex), and quantification was performed using known external standards at multiple concentrations.

### Calculations and statistics

Specific growth rate (SGR) of fish was calculated according to the following equation:

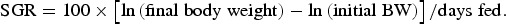


Feed conversion ratio (FCR) was calculated as:

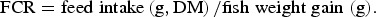

Daily feed intake per fish was calculated on a DM basis as:

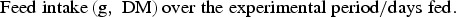

Data were analysed by a one-way ANOVA using the general linear model (PROC GLM) and, when appropriate, by the χ^2^ test in SAS 9.4 (SAS Institute, Inc.). Tukey's honestly significant difference (HSD) test was performed for *post hoc* analysis. In addition, orthogonal contrasts for linear, quadratic and cubic effects of the diets were determined using the ESTIMATE statement in PROC GLM. The OR was calculated (PROC LOGISITIC) as the ratio of the odds of bacterial translocation (PCR-positive) when SBMIE was present to the odds of bacterial translocation when SBMIE was absent. Data from histological evaluation and plasma lactulose and DNA level were not normally distributed after log 10-transformation; and thus the analysis was performed using a non-parametric Kruskal–Wallis test by ranks followed by the Wilcoxon rank-sum test. The level of significance was set at *P* < 0·05.

## Results

### Growth rate and feed conversion

Feed intake, growth rate and FCR are given in Table 3. There was a significant reduction in feed intake with the inclusion of 37·5 % SBM in the diet compared with the FM diet (*P* = 0·04). Adding SBM to the diets had a significant effect on FCR (*P* < 0·001). Fish fed the FM control diet had significantly higher FCR than those fed SBM-containing diets. Fish fed the 37·5 % SBM diet had the lowest FCR, while the fish fed the 25 % SBM diet had lower FCR than those fed the 12·5 % SBM diet. There were no differences among the diets for final weight and specific growth rate (SGR).

**Table 3.** Feed intake and growth performance of rainbow trout (*Oncorhynchus mykiss*) fed the experimental diets for 31 d (Mean values with pooled standard errors; *n* 3)

	FM	SBM 12·5 %	SBM 25 %	SBM 37·5 %	sem	*P*
Initial weight (g)	235·8	236·2	235·2	235·2	2·51	0·91
Final weight (g)	334·3	327·9	344·2	330·5	9·13	0·13
Specific growth rate (%)	1·1	1·1	1·2	1·1	0·11	0·14
Feed intake (g/d)	2·77^a^	2·23^a,b^	2·47^a,b^	2·16^b^	0·263	0·04
Feed conversion ratio	0·87^a^	0·75^b^	0·70^b^	0·70^b^	0·021	<0·0001

FM, fish meal; SBM, soyabean meal.

^a,b^ Mean values within a row with unlike superscript letters were significantly different (*P*<0·05).

### Morphological evaluation of the distal intestine

**Fig. 1.**
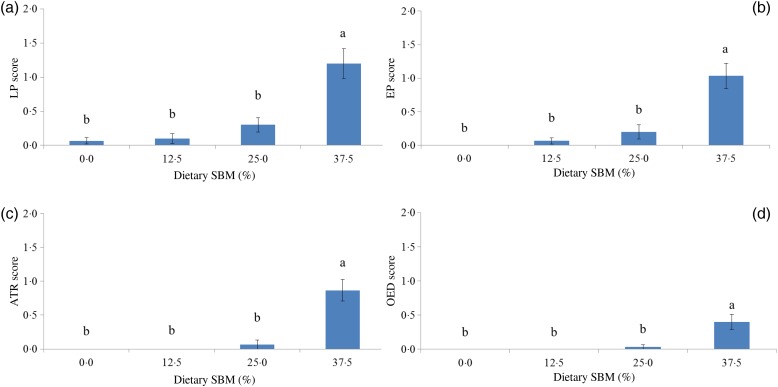
Morphological evaluation of the distal intestine of rainbow trout (*Oncorhynchus mykiss*) fed a fish meal-based diet and three experimental diets containing soyabean meal (SBM) at levels of 12·5, 25 and 37·5 %. Changes in the leucocyte infiltrates in the lamina propria and submucosa (LP) (a); changes in the epithelium (EP) (b); atrophy of the intestinal folds (ATR) (c); and accumulation of protein-rich fluid in the lamina propria defined as oedema (OED) (d). Values are means (*n* 15), with standard errors represented by vertical bars. ^a,b^ Mean values with unlike letters were significantly different (*P* < 0·05).

**Fig. 2.**
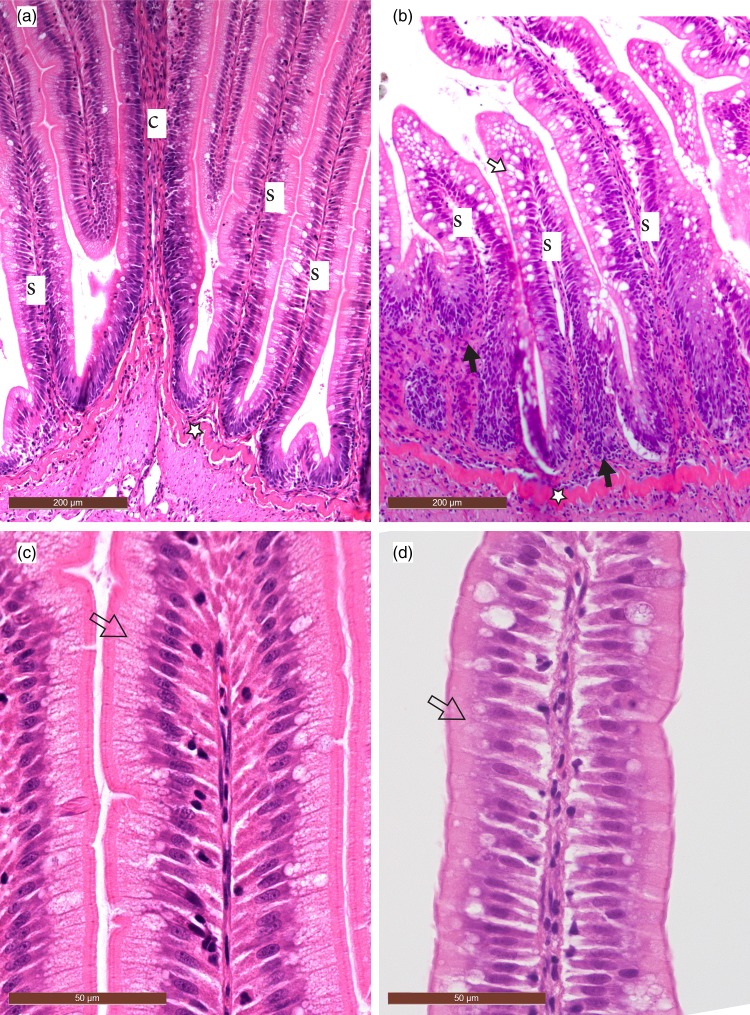
Morphology of the distal intestine in rainbow trout (*Oncorhynchus mykiss*) stained with haematoxylin and eosin (H&E) at low and high magnifications, respectively. Bars indicate the actual magnification. (a) Low magnification: fish meal control (non-soyabean meal-induced enteritis; non-SBMIE) – a normal intestine without subepithelial infiltrates of inflammatory leucocytes and slender simple (s) and complex (c) folds outlined by regular, high and finely vacuolated columnar epithelial cells. (b) Low magnification: soyabean meal 37·5 % (SBMIE) – an inflamed intestine with atrophy of complex and simple folds and heavy infiltration of the subepithelial intestinal mucosa (black arrows) with inflammatory leucocytes and proliferation of fibroblasts, indicating a subacute state of inflammation. Note the many large, clear vacuoles in the epithelium (white arrow) which is probably due to the proliferation of goblet cells. The point stars on (a) and (b) indicate the stratum compactum. (c) High magnification: fish meal control (non-SBMIE) – a normal, high columnar epithelium with a finely vacuolated supranuclear cytoplasm (arrow) and a distinct brush border. (d) High magnification: soyabean meal 37·5 % (SBMIE) – the epithelial cells have a denser cytoplasm (arrow), are lower in height and lack the finely vacuolated supranuclear zone seen in the normal tissue, although some clear and quite large intracytoplasmic vacuoles can be seen, probably due to the presence of goblet cells. The brush border is less distinct compared with the control.

The mean scoring of morphological parameters is shown in Fig. 1. Diets containing 12·5 and 25 % SBM showed minor morphological changes, but were not statistically different from the FM control diet. Fish fed the 37·5 % SBM diet showed significantly increased changes in all morphological parameters (*P* < 0·05). These changes were characterised by reduced supranuclear vacuoles and height of epithelial cells, reduced height of intestinal folds (atrophy) and widening of the lamina propria due to oedema and increased numbers of leucocytes (Table 2; Fig. 2). Histological evaluation revealed SBMIE in ten fish with varying degrees of morphological changes from the group fed 37·5 % SBM and in one fish displaying a moderate SBMIE from the group fed 25 % SBM.

### PCR-denaturing gradient gel electrophoresis analysis

**Fig. 3.**
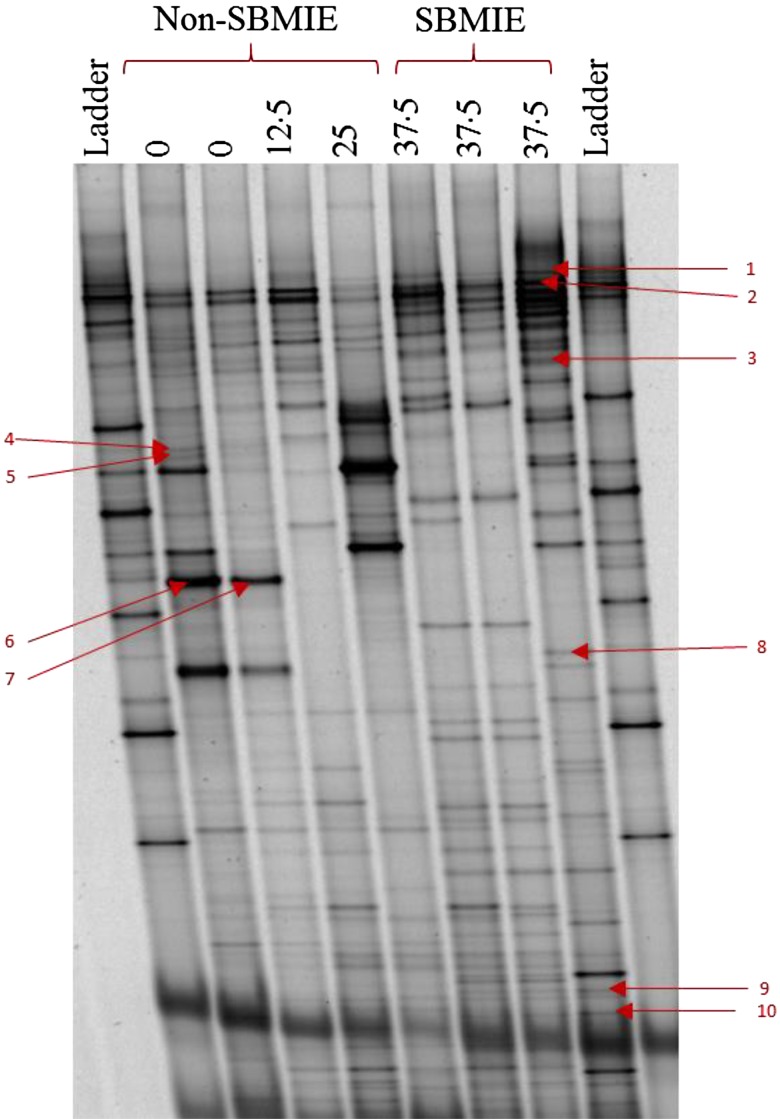
Denaturing gradient gel electrophoresis (DGGE) profile of 16S rDNA amplicons from the plasma of PCR positive rainbow trout (*Oncorhynchus mykiss*) fed diets with different soyabean meal (SBM) inclusion levels: 0, 12·5, 25 and 37·5 %. Bands 1–10 are excised DGGE bands used for sequence analysis. See Table 4 for details of each band's identification. SBMIE, SBM-induced enteritis.

A total number of seven plasma samples were positive for bacterial 16S rDNA detection. DGGE analysis of the PCR products from these samples is presented in Fig. 3. The results from sequence analysis of excised bands from the DGGE gel are shown in Table 4. There was a total number of fourteen bands sequenced, of which four were short sequences (138–141 bp) and represented unspecific amplification (not shown), six represented *Staphylococcus* spp., while the remaining four bands were identified by BLAST (Basic Local Alignment Search Tool) as different *Escherichia coli* strains.

**Table 4.** Identification of denaturing gradient gel electrophoresis bands obtained from plasma based on 16S rDNA sequencing of the V3 region

Band no.	Sequence length (bp)	Identification by BLAST	Homology (%)	GenBank accession no.
1	161	*Staphylococcus* sp. *ECBMB15*	100	KJ425241.1
2	161	*Staphylococcus* sp. *ECBMB11*	100	KJ425241.1
3	161	*Staphylococcus cohnii strain BDA10*	94	HQ641334.1
4	161	*Escherichia coli strain E417-1*	99	KJ477010.1
5	163	*Escherichia coli strain G3T64*	94	GU646103.1
6	161	*Escherichia coli strain E417-1*	99	KJ477010.1
7	161	*Escherichia coli strain E417-1*	99	KJ477010.1
8	162	*Staphylococcus* sp. *ECBMB15*	97	KJ425241.1
9	162	*Staphylococcus* sp. *ECBMB15*	99	KJ425241.1
10	161	*Staphylococcus* sp. *ECBMB15*	100	KJ425241.1

BLAST, Basic Local Alignment Search Tool.

### Plasma levels of intestinal permeability markers

Increasing dietary SBM inclusion resulted in non-significant changes in plasma endotoxin and total genomic DNA concentration (Table 5). None of the sugar markers could be detected in plasma from fish fed control and experimental diets on day 28 before the addition of dietary sugar markers. Variation in plasma sugar marker concentration was generally large among individuals; however, the variation was found to be somewhat lower for l-rhamnose (Table 5). Plasma sucralose levels were below the detection limit in many individuals and for all fish fed the 12·5 and 25 % SBM diets. The plasma levels of sucralose were not significantly different between FM- and 37·5 % SBM-fed fish. Plasma l-rhamnose levels were, however, significantly reduced in the group fed 37·5 % SBM (*P* = 0·01). The variation was more prominent for lactulose and the plasma level of this sugar was not significantly different between FM-fed and 37·5 % SBM-fed fish. Plasma L:R and sucralose:l-rhamnose (S:R) ratios in 37·5 % SBM-fed fish were not different from those in the control FM fish. Mean plasma d-lactate concentration was significantly higher in 37·5 % SBM-fed fish compared with the other groups (*P* < 0·0001) (Table 5). The ESTIMATE statement revealed a linear increase in the plasma level of d-lactate with increasing level of SBM inclusion in the diet (*P* < 0·0001) while no linear, quadratic and cubic effects were observed for other permeability markers.

**Table 5.** Effect of diets on the level of intestinal permeability markers in plasma (Mean values with pooled standard errors;  fifteen fish per diet)

	FM	SBM 12·5 %	SBM 25 %	SBM 37·5 %	sem	*P*
Permeability markers						
Endotoxin (EU/ml)	0·09	0·08	0·09	0·08	0·019	0·621
DNA concentration (μg/ml)*	1·30	1·09	1·14	1·06	0·296	0·263
d-Lactate (μg/ml)	6·19^b^	7·01^b^	6·80^b^	8·69^a^	1·288	<0·0001
Sugar markers (μg/ml)						
Lactulose†	9·20^a,b^	2·00^b^	2·89^b^	10·26^a^	3·949	0·012
Sucralose	0·71	ND	ND	1·19	0·951	0·421
l-Rhamnose	15·65^a,b^	17·09^a^	14·86^a,b^	10·25^b^	3·648	0·013
L:R†	0·54^a,b^	0·16^b^	0·22^b^	1·70^a^	1·013	0·010
S:R	0·04	ND	ND	0·08	0·041	0·189

FM, fish meal; SBM; soyabean meal; EU, endotoxin units; ND, not detected; L:R, lactulose:l-rhamnose ratio; S:R, sucralose:l-rhamnose ratio.

^a,b^ Mean values within a row with unlike superscript letters were significantly different (*P* < 0·05).

* Genomic DNA measured after extraction.

† The Kruskal–Wallis test was performed for this parameter.

### Relationship between soyabean meal-induced enteritis, plasma permeability markers and PCR

Differences in plasma endotoxin and genomic DNA concentration were found to be insignificant between SBMIE and non-SBMIE fish (Table 6). Three of eleven fish with SBMIE were shown to be PCR-positive (28 %) while this rate tended to be lower in non-SBMIE group, with only four of forty-nine (8 %) being PCR-positive (*P* = 0·07). Three of seven PCR-positive fish (43 %) had also SBMIE while this ratio was only eight of fifty-three (15 %) in PCR-negative fish (*P* = 0·074) (Table 6). The OR of positive PCR in fish with SBMIE was 4·5 (95 % CI 1·92–10·34) relative to the non-SBMIE fish (*P* = 0·0005). The plasma L:R ratio was found to be higher in fish with SBMIE than that in non-SBMIE fish, but variation was high in this category (Table 6). Plasma l-rhamnose was, however, reduced in the SBMIE group (*P* = 0·03). Fish with SBMIE demonstrated a higher plasma d-lactate level than non-SBMIE fish (*P* < 0·05). A positive but weak correlation was also found between the plasma level of d-lactate and degree of SBMIE (*n* 60, *F* = 24·6, *r*^2^ 0·30, *P* < 0·001).

**Table 6.** PCR results and plasma levels of intestinal permeability markers in soyabean meal (SBM)-induced enteritis (SBMIE) and non-SBMIE groups of rainbow trout (*Oncorhynchus mykiss*) (Mean values with pooled standard errors)

Group	SBMIE*	Non-SBMIE	sem	*P*
*n*	11	49		
PCR-positive samples	3	4		
Genomic DNA (μg/ml)	0·92	1·04	0·150	0·52
Endotoxin (EU/ml)	0·08	0·07	0·011	0·71
Sugar markers (μg/ml)				
L:R†	1·06^a^	0·47^b^	0·325	0·02
S:R	0·08	0·04	0·031	0·23
l-Rhamnose	11·01^b^	15·24^a^	1·969	0·03
d-Lactate	8·91^a^	6·77^b^	0·490	<0·0001

EU, endotoxin units; L:R, lactulose:l-rhamnose ratio; S:R, sucralose:l-rhamnose ratio.

^a,b^ Mean values within a row with unlike superscript letters were significantly different (*P* < 0·05).

* Includes ten fish from the 37·5 % SBM-fed group and one fish from the 25 % SBM-fed group.

† The Kruskal–Wallis test was performed for this parameter.

## Discussion

The growth of fish fed increasing levels of SBM was comparable with that of the fish fed FM, as the SGR did not change in response to SBM inclusion. FCR gradually decreased as a result of increased SBM inclusion, which is not consistent with previous studies^(^^42^^,^^43^^)^. Lower FCR in this experiment may be due to the lower feed consumption by SBM-fed fish or a reduced passage rate of the feed and thus increasing the time for nutrient uptake in these fish. While increased inflammatory response was evident in the group fed 37·5 % SBM, no adverse effect was observed on fish growth, which is in accordance with previous studies^(^^44^^)^. Moreover, the histological evaluation revealed a moderate degree of inflammation in fish fed 37·5 % SBM, which may further indicate that rainbow trout seems to be less sensitive to SBM than Atlantic salmon^(^^45^^)^. A higher number of fish with SBMIE as a result of increased SBM inclusion is also consistent with previous reports^(^^6^^)^.

The relationship between gut barrier disturbance and the development of SBMIE is not yet known in fish. In this experiment, we hypothesised that SBMIE is associated with increased intestinal permeability which further promotes translocation of micro-organisms and/or their products into the bloodstream. In humans and mammals, bacterial translocation from the lumen into the circulation has been reported in subjects with intestinal inflammation^(^^46^^,^^47^^)^. Translocation of bacteria into the blood indicates a loss or impaired gut barrier function which may occur under different conditions. Under homeostatic conditions, some bacterial translocation may occur but is cleared by the organism's immune system^(^^48^^)^. The rate of normal bacterial translocation has been reported to be in the range of 5–10 % in humans^(^^49^^)^ and 10–20 % in animals^(^^50^^)^. When gut homeostasis is disturbed, however, a rise in the rate of bacterial translocation may occur^(^^49^^)^. In fish, the normal rate of bacterial translocation and its association with SBMIE is not known; however, there is evidence that gut bacteria translocate into enterocytes in larvae, fry and adult fish^(^^51^^)^. In this study, we used PCR-DGGE to detect and identify circulating bacterial DNA in fish. Our results indicate that the detection of bacterial DNA in plasma is more frequently associated with the incidence of SBMIE (OR 4·5) which may suggest a link between SBMIE and bacterial translocation. Disruption of the gut barrier has shown to be associated with bacterial translocation in a number of human studies^(^^27^^,^^33^^,^^34^^)^. The PCR-DGGE analyses in the present experiment identified several strains of two species of bacteria, *Staphylococcus* spp. and *E. coli*, and the latter has been known as a common translocating bacteria in humans^(^^52^^)^. Some strains of both *E. coli* and *Staphylococcus* spp. are known to cause infection, but PCR only detects bacterial DNA and does not differentiate between dead or living bacteria.

In this experiment, an increasing level of SBM inclusion and the occurrence of SBMIE did not increase plasma levels of endotoxins compared with FM-fed and non-SBMIE fish. The reason could be that endotoxins are cleared rapidly by the mucosal immune system, while trying to cross the epithelium under SBMIE.

Increased intestinal permeability, reflected by elevated urinary levels of sugar markers, has been reported in humans with impaired intestinal barrier integrity^(^^17^^,^^18^^)^. A high urinary or plasma L:R ratio is often used as an indication of small-intestinal hyperpermeability in mammals. Our results, however, do not show increased plasma L:R and S:R ratios as a result of increased SBM inclusion and revealed large variation among individuals in all dietary groups (Table 5). The L:R ratio was significantly increased in the SBMIE group, while the increase in the S:R ratio was not significant, presumably because of the small number of observations for sucralose (*n* 6). The elevated L:R ratio in the SBMIE group is in accordance with previous findings in humans^(^^18^^)^ and may indicate that intestinal inflammation is associated with DI hyperpermeability; however, the variation was also found to be large for this parameter. Sugar markers were added to the diets; consequently, any variation in feed intake could reflect differences in the absorption of these molecules. Contrary to humans and model animals, the group feeding system is practised to feed the fish without monitoring the individual consumption of diets. Thus it is likely that individual fish used for plasma sugar analysis had consumed unequal amounts of these molecules before sampling. Based on these results, it seems that feed-added markers may not be suitable for the evaluation of intestinal permeability in fish that are group-fed. In this experiment, however, the SBMIE group had significantly lower plasma l-rhamnose levels which may be due to the epithelial changes as a result of SBMIE, with consequent adverse effect on l-rhamnose absorption. Most of the fish (ten of fifteen) from the group fed 37·5 % SBM had SBMIE which consequently resulted in a reduced mean of plasma l-rhamnose level in this group. A reduction in excretion levels of l-rhamnose as a result of increased gut permeability has been reported previously in human subjects^(^^53^^)^. The possible explanation is that the intestinal absorptive area may be reduced in response to inflammation which in turn can decrease the uptake of l-rhamnose into the bloodstream^(^^54^^)^.

Increased plasma levels of d-lactate have been shown in humans and model animals with intestinal barrier injury^(^^55^^–^^57^^)^. In fish with SBMIE, plasma d-lactate concentrations increased compared with the non-SBMIE fish, which suggests that increased intestinal permeability may have occurred as a result of inflammation. Furthermore, an increased degree of SBMIE showed a weak but significant correlation with plasma levels of d-lactate. This may suggest that other factors than SBMIE can also contribute to the increased plasma level of d-lactate. It has been shown, for example, that the increase in bacterial fermentation in the lumen as a result of bacterial overgrowth or carbohydrate malabsorption may also increase the level of d-lactate in the circulation independent of inflammation^(^^58^^,^^59^^)^. We also found a significant linear increase in plasma d-lactate as a result of increased SBM inclusion level, which raised the question whether there is a dietary effect on plasma d-lactate level independent of inflammation. However, we did not observe an increased d-lactate concentration in DI content from fish fed 37·5 % SBM compared with the control group (data not shown), which may suggest that there is a relationship between SBMIE and increased gut permeability.

In conclusion, our results show that SBMIE resulted in increased plasma levels of d-lactate and an increased incidence of bacterial translocation. The plasma lactulose:l-rhamnose ratio was found to increase in fish with SBMIE; however, the analysis revealed a large variation among individuals, probably due to unequal feed consumption. This indicates that feed added markers are less reliable under the group-feeding strategy. Based on our results, d-lactate and PCR-based detection of bacteria are more suitable estimates for *in vivo* permeability assessment under SBMIE conditions in rainbow trout.
